# The Sacral Frame Technique: A Novel Trans/Extrasacral Approach for Giant Sacral Schwannomas Resection with Sacropelvic Biomechanics Preservation

**DOI:** 10.3390/jcm14238511

**Published:** 2025-11-30

**Authors:** Carlo Brembilla, Pietro Paolo Cotrufo, Ali Baram, Mario De Robertis, Laura Samà, Gabriele Capo, Donato Creatura, Maurizio Fornari, Federico Pessina, Ferdinando Carlo Maria Cananzi

**Affiliations:** 1 Department of Neurosurgery, IRCCS Humanitas Research Hospital, Via Alessandro Manzoni 56, 20089 Rozzano, MI, Italy; pietro.cotrufo@humanitas.it (P.P.C.); ali.baram@humanitas.it (A.B.); mario.derobertis@humanitas.it (M.D.R.); gabriele.capo@humanitas.it (G.C.); donato.creatura@humanitas.it (D.C.); maurizio.fornari@humanitas.it (M.F.); federico.pessina@hunimed.eu (F.P.); 2 Department of Biomedical Sciences, Humanitas University, Via Rita Levi Montalcini 4, 20090 Pieve Emanuele, MI, Italy; ferdinando.cananzi@hunimed.it; 3 Sarcoma, Melanoma and Rare Tumors Surgery Unit, IRCCS Humanitas Research Hospital, Via Manzoni 56, 20089 Rozzano, MI, Italy; laura.sama@humanitas.it

**Keywords:** sacral schwannoma, giant schwannoma, sacropelvic biomechanics, sacral tumor, sacral surgical approach, sacropelvic fixation

## Abstract

**Background:** Giant sacral schwannomas present a significant surgical challenge, often requiring extensive resections that compromise neurological function and sacropelvic biomechanics. Conventional approaches frequently necessitate sacral bone sacrifice, resulting in the deafferentation of key pelvic stabilizers and subsequent long-term functional deficits. This study introduces the novel single-posterior “Sacral Frame Technique,” designed to preserve the lateral sacral bone margin and optimize functional reconstruction. **Methods:** We describe the surgical technique and report on a case of a 55-year-old female with a giant sacral schwannoma extending into the spinal canal and presacral space. The resection was performed via a combined trans-sacral and extrasacral approach, employing an intralesional piecemeal strategy to maintain the lateral sacral bone margin. The gluteus maximus muscles, along with the sacrotuberous and sacrospinous ligaments, were meticulously reattached to their natural insertion sites on the preserved bone. Clinical and radiological outcomes were evaluated at six months post-operatively. **Results:** Complete tumor resection was achieved without post-operative neurological deficits or sphincter dysfunction. The patient achieved early mobilization, returned to pre-operative activity levels, and showed no evidence of sacropelvic instability at the six-month follow-up. Post-operative imaging confirmed complete tumor clearance and the structural integrity of the preserved sacral bone margin. **Conclusions:** The “Sacral Frame Technique” offers a potential strategy for the safe and effective resection of giant sacral schwannomas. By prioritizing the preservation of the lateral sacral bone margin, the technique facilitates the anatomical reattachment of pelvic stabilizers, potentially mitigating long-term biomechanical deficits. Further studies with larger cohorts are warranted to fully validate these findings and establish the broader applicability of this bone-preserving approach.

## 1. Introduction

Sacral schwannomas, though relatively infrequent, present a significant surgical challenge, particularly when attaining giant dimensions and extending into the spinal canal and adjacent pelvic structures. The management of these large tumors necessitates a meticulous and multidisciplinary approach, and complete surgical resection remaining the gold standard of treatment due to their benign nature [1,2,3]. However, the tumors’ proximity to critical neurovascular structures presents considerable challenges. Consequently, achieving complete tumor resection while simultaneously preserving neurological function and the biomechanical integrity of the sacropelvic complex remains the surgeon’s paramount concern [4,5].

Traditional surgical strategies for giant sacral schwannomas often involve extensive dissection and significant patient morbidity, often employing combined anterior and posterior approaches for adequate exposure and resection [1,2,3,4,5]. Furthermore, wide resections often necessitate debilitating sacropelvic fixations [6]. Although significant advances in surgical techniques, intraoperative imaging modalities, robotic assistance, and sophisticated neurophysiological monitoring have led to improved outcomes [7,8,9,10,11], the inherent complexity of these cases still necessitates the exploration of novel and potentially less morbid surgical strategies.

This article describes our innovative single-posterior approach, the “Sacral Frame Technique,” for the resection of giant sacral schwannomas with massive presacral extension and spinal canal invasion. Our methodology employs a trans-sacral access via laminectomy for the initial decompression of the intraspinal component of the tumor. Subsequently, the approach is expanded to the left sacral margin, requiring careful detachment of the medial insertion of the gluteus maximus muscle, the sacrotuberous and sacrospinous ligaments, and the coccygeus muscle for complete exposure of the presacral pelvic cavity. Critically, our technique emphasizes the meticulous preservation of the lateral sacral bone margin, allowing for precise reattachment of these soft-tissue structures, and thereby ensuring optimal functional reconstruction and maintenance of sacropelvic musculoskeletal biomechanical integrity.

## 2. Case Presentation

**Figure 1:**
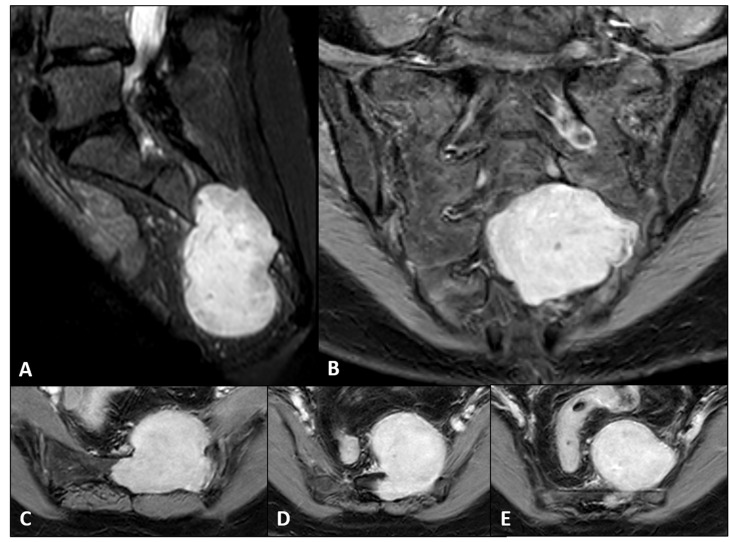
Preoperative imaging demonstrating the giant sacral schwannoma. (**A**) Sagittal contrast-enhanced T1-weighted MRI showing the cranial-caudal extent and significant intraspinal and presacral component. (**B**) Coronal view illustrating the lateral spread and foraminal involvement. (**C**–**E**) Axial views highlighting the extensive presacral mass and its relationship to the lower sacral segments.

A 55-year-old female with no significant past medical history presented with the onset of sacral pain. Diagnostic workup, initiated with lumbar spine MRI, which revealed a large (≈6.8 cm), well-defined, oval mass centered at the S3 level, extending superiorly to S2 and involving the left S2–S3 foramina. The lesion was predominantly situated anteriorly in the presacral space and pelvic cavity. Subsequent contrast-enhanced sacral MRI (Figure 1) confirmed these findings, demonstrating a homogeneously and diffusely enhancing mass, raising suspicion for a schwannoma over the initial consideration of a chordoma. A lumbosacral CT scan corroborated the MRI data, identifying a cystic mass at S2 with non-erosive expansion of the left S3–S4 foramina, highly suggestive of slow growth.

Following a comprehensive multidisciplinary consultation with our specialized Rare Tumor Board, comprising general surgeons specializing in rare tumors, radiation oncologists, medical oncologists, expert pathologists, and neurosurgeons, a CT-guided biopsy and a contrast-enhanced chest and abdominal CT scan were recommended to rule out metastatic disease. The CT-guided biopsy subsequently confirmed the diagnosis of a schwannoma.

Neurological examination at presentation revealed no deficits in higher cognitive functions, cranial nerves, or lower limb motor and sensory function, with the patient exhibiting a normal gait and intact sphincter control. Based on the established diagnosis and the collective recommendation of the multidisciplinary team, surgical excision was indicated. A Gross Total Resection (GTR) was performed using the “Sacral Frame Technique” using an intralesional, piecemeal technique.

The post-operative course was uneventful. The patient mobilized independently, and the urinary catheter was removed on the first post-operative day. A post-operative lumbar-sacral spine MRI confirmed complete lesion excision on the second post-operative day. No sensory or motor disturbances were observed, and sphincter control remained intact. The patient was discharged home on the fifth post-operative day without any neurological deficits.

**Figure 2:**
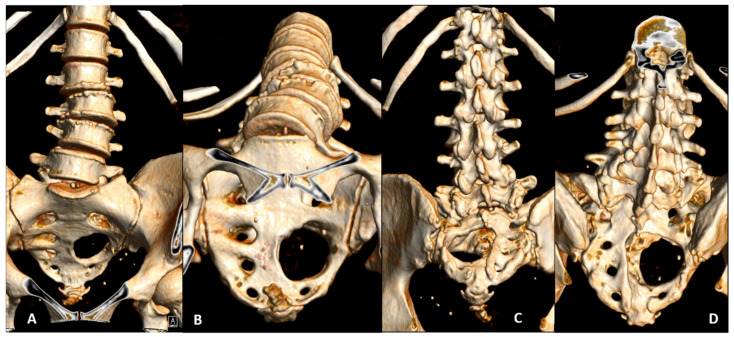
Three-month postoperative Photon-counting CT (PCCT) 3D reconstruction confirming Sacral Frame integrity and stable spinopelvic biomechanics. PCCT was utilized for its superior spatial resolution, enabling detailed visualization and comprehensive analysis of the bone architecture and residual sacral stability. (**A**) Antero-posterior view; (**B**) Caudo-cranial view; (**C**) Postero-anterior view; (**D**) Cranio-caudal view.

**Figure 3:**
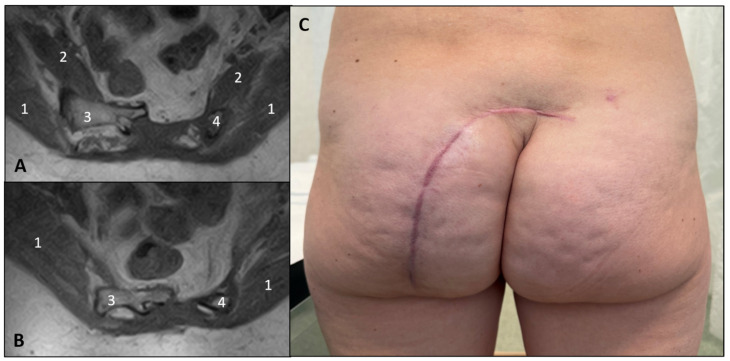
(**A**,**B**) Post-operative Magnetic Resonance Imaging (MRI) at six months. Axial T1-weighted MRI cuts ((**A**): cranial, (**B**): caudal) confirm complete lesion excision with no evidence of residual or recurrent tumor. The images effectively demonstrate the successful preservation of adjacent soft tissues and the biomechanical structures. Specifically highlighted are: (1) the symmetrical and preserved bulk of the gluteus maximus muscles; (2) the intact piriformis muscles; (3) the sacral body and right side of the sacrum; and (4) the preserved left lateral sacral margin (the ‘frame’ branch). (**C**) The patient’s posterior low back and gluteal region at the six-month follow-up show the well-healed arciform incision line and the symmetrical shape and trophism of the gluteal muscles, indicating successful preservation of functional anatomy and optimal cosmetic outcome.

At three months, a Photon-counting CT scan (PCCT) of the sacrum (Figure 2) confirmed the integrity of the preserved sacral bone margins. At the six-month clinical follow-up, a contrast-enhanced MRI (Figure 3) demonstrated no evidence of residual tumor or recurrence, with perfect anatomical symmetry and comparable trophism of the gluteal muscles. The patient remained neurologically intact, exhibited a normal gait (even on stairs), and had resumed jogging activities.

## 3. Surgical Technique

The surgical resection of the giant sacral schwannoma was performed through a collaborative approach involving the Neurosurgery and General Surgery Units for Rare Tumors. The procedure was conducted with the patient in the prone position, under general anesthesia and continuous Intraoperative Neurophysiological Monitoring (IONM). Preoperative CT imaging was performed using a Medtronic O-arm™ Navigation System (Littleton, MA, USA) to guide the approach. A detailed schematic of the surgical steps is provided in Table 1.

**Figure 4:**
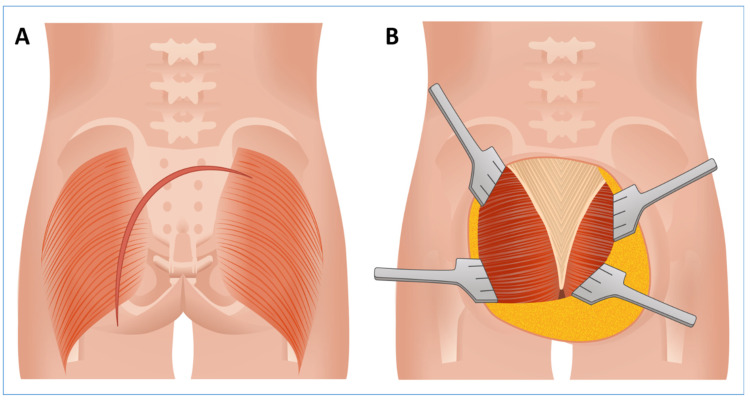
Intraoperative views of the surgical incision and initial soft tissue dissection. (**A**) Skin Incision: the placement and design of the arciform incision. (**B**) Soft Tissue Exposure: the elevated fasciocutaneous flap revealing the bilateral gluteus maximus muscles (left side predominant) and the central thoracolumbar fascia, defining the surgical access area.

**Figure 5:**
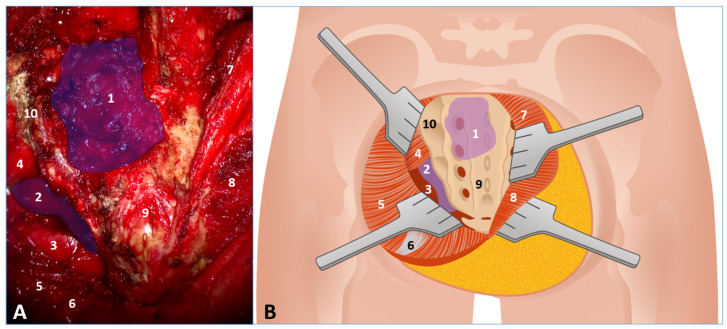
Surgical Exposure for the “Sacral Frame Technique.” (**A**) Intraoperative microscopic view of the surgical field following initial dissection. (**B**) Corresponding stylized anatomical illustration of the surgical approach and relevant structures. The images display the full exposure of the sacrum and the pelvic presacral cavity, highlighting the initial step of the bone-preserving technique. Key anatomical structures and surgical landmarks are numbered as follows: 1. Trans-sacral component of the schwannoma (intraspinal/intrasacral tumor extension); 2. Extra/presacral extension of the schwannoma (tumor mass extending into the pelvic cavity); 3. Mesorectum (dissected to expose the anterior presacral space); 4. Piriformis muscle (displaced cranially by the tumor mass); 5. Detached and reflected left gluteus maximus; 6. Detached and reflected sacrotuberous and sacrospinous ligaments; 7. Detached and reflected right longissimus thoracis and iliocostalis lumborum muscles; 8. Right gluteus maximus muscle; 9. Mid and lower sacrum; 10. Posterior Inferior Iliac Spine (PIIS).

A left gluteal arc-shaped incision with inferomedial concavity extended towards the sacrum (Figure 4A). Following the preparation of the skin and subcutaneous flaps (Figure 4B), a midline incision of the thoracolumbar fascia was performed. The paravertebral muscles (longissimus thoracis and iliocostalis lumborum) were bilaterally dissected and mobilized. On the left side, the dissection was extended to meticulously mobilize en bloc the gluteus maximus muscle, the sacrotuberous and sacrospinous ligaments, and the coccygeus muscle. This provided complete exposure of the left lateral margin of the sacrum, from the greater sciatic notch (at the level of the posterior inferior iliac spine) caudally to the coccyx, without requiring anococcygeal ligament transection. The piriformis muscle and the superior gluteal artery were identified, both displaced cranially by the tumor mass. Subsequently, blunt dissection exposed the lower endopelvic/presacral component of the lesion, located just posterior to the mesorectum, thereby preparing the surgical field for the neurosurgical phase (Figure 5).

Under microscopic magnification, the neurosurgical dissection began by identifying the sacral bone and the left paramedian extrasacral portion of the tumor. At the level of the sacral lamina, tumor-induced erosion was evident as bony discontinuity. A sacral laminectomy was carefully performed to expose the intracanal component of the mass and the cauda equina. The tumor was meticulously dissected and separated from the surrounding neural structures. The afferent nerve roots associated with the tumor were identified, mapped through IONM, coagulated, and sectioned. The lesion was then progressively coagulated and debulked from within its intracanal component. This debulking facilitated the subsequent dissection of the entire mass. Following the removal of the intracanal portion, debulking then continued towards the extracanal presacral intrapelvic component via the median trans-sacral approach.

The partially emptied mass was then addressed through the left extrasacral window. Here, the efferent nerve root of the tumor was identified, mapped, coagulated, and sectioned. The extrasacral window also facilitated meticulous dissection of the lesion from the left internal iliac vein and its afferent vessels (located in the supero-antero-lateral portion of the extrasacral intrapelvic surgical cavity), enabling complete resection of the lesion. Following complete tumor resection, meticulous hemostasis was achieved. A drain was placed within the surgical cavity.

The tendinous-ligamentous complex, encompassing the proximal tendon of the gluteus maximus muscle, the sacrotuberous, and sacrospinous ligaments, was firmly reattached to the lateral margin of the sacrum—bony rim measuring approximately 17 mm in width and 10 mm in thickness—at their natural insertion site (left inferior lateral angle). A non-absorbable braided suture, Ethicon Excel (Ethicon Inc.-Somerville, NJ, USA), size 2-0, was used for fixation.

The wound was closed in a layered fashion, including closure of the thoracolumbar fascia, followed by quilting of the subcutaneous flaps and skin closure. Throughout the procedure, IONM showed no changes.

## 4. Discussion

Giant sacral schwannomas, despite their rarity, present a formidable surgical challenge due to their potential for extensive growth, often involving the sacral bone, the spinal canal, and adjacent pelvic structures. Complete surgical resection remains the cornerstone of treatment for these benign tumors [1,2,3]. However, achieving this goal in large lesions, particularly those with significant intraspinal and extensive presacral components in close proximity to critical neurovascular structures, poses considerable difficulties [4,5].

Traditional surgical approaches for such giant tumors have frequently necessitated combined anterior and posterior routes to gain adequate exposure and facilitate complete resection [1,2,3,4,5]. These extensive dissections often carry significant patient morbidity, including the frequent need for debilitating sacropelvic fixations, especially when a substantial portion of the sacrum must be sacrificed for tumor access and removal [5,6,7]. Nevertheless, advancements in surgical techniques, including the use of intraoperative navigation, robotic assistance, and minimally invasive approaches, are increasingly being explored to enhance precision and promote greater preservation of the surrounding anatomical structures [7,8,9,10,11].

Current literature on functional outcomes following sacral surgery primarily focuses on two aspects: neurological status (with emphasis on preserving motor and sensory function mediated by the sciatic, femoral, and sacral nerves) and the stability of the pelvic ring (evaluated by the validity of reconstructive fixation constructs following extensive resection) [12,13,14]. While the maintenance of neural integrity and pelvic stability are undoubtedly critical, the residual functional biomechanics related to the muscular and ligamentous structures of the pelvis are frequently underestimated, even when neurological outcomes and pelvic stability appear favorable.

The resection of the lateral sacral bone margin, often required to access the pelvic presacral cavity and achieve complete removal of giant schwannomas, can lead to the deafferentation of crucial pelvic stabilizers. The medial insertion of the gluteus maximus muscle and the sacrotuberous and sacrospinous ligaments normally attach to this lateral margin. When this vital attachment site is compromised or resected, reattachment to their natural anatomical position becomes challenging or impossible. Consequently, surgeons often resort to suturing these structures to the midline or other less biomechanically advantageous locations [4,5,15].

This altered reattachment can lead to significant functional impairment. The gluteus maximus muscle, a primary hip extensor crucial for propulsive force during gait and stair climbing, may suffer from atrophy and weakness due to suboptimal reinsertion, resulting in noticeable functional deficits [16,17].

Furthermore, the sacrotuberous and sacrospinous ligaments play a vital role in pelvic stability, acting as natural restraints against excessive posterior pelvic tilt and sacral nutation. Disruption of their natural attachments can lead to dynamic sacroiliac instability, potentially affecting the biomechanics of the hip and trunk movements. The intricate interplay between sacral motion and lower limb function underscores the importance of maintaining pelvic stability [18,19,20,21].

The functional outcome is further complicated by the sacrotuberous ligament’s intimate connection to the proximal hamstring tendon. A significant portion of the posterior thigh hamstring fibers insert onto this ligament, extending beyond the ischial tuberosity. Compromising the integrity of the sacrotuberous ligament during surgery can therefore directly impact the biomechanics and strength of the hamstring muscles, contributing to weakness and altered gait patterns. The literature emphasizes this crucial role in stabilizing the sacroiliac joint and its interaction with the biceps femoris muscle, suggesting that altered tension or damage can have far-reaching effects on spinal load and overall lower limb function [18,19,20,21].

In contrast to traditional approaches, our novel single posterior “Sacral Frame Technique” prioritizes the meticulous preservation of the lateral sacral bone margin. The benign nature of giant sacral schwannomas, which allows for intralesional piecemeal resection, enables a combined trans-sacral approach with an extrasacral window for access to the pelvic cavity. By maintaining this crucial anatomical landmark, our technique allows for the precise reattachment of the gluteus maximus muscle, the sacrotuberous, and sacrospinous ligaments to their natural insertion sites. This preservation aims to ensure optimal functional reconstruction and the maintenance of sacropelvic musculoskeletal biomechanics, potentially mitigating the long-term functional deficits associated with more radical resections.

The successful outcome in our presented case—with the patient achieving early mobilization, maintaining neurological function and sphincter control, and returning to pre-operative activity levels with no evidence of instability at six-month follow-up—strongly supports the potential benefits of this bone-preserving approach.

Further studies with larger cohorts and longer follow-up periods are necessary to validate the long-term functional outcomes and biomechanical advantages of the Sacral Frame Technique in comparison to traditional surgical strategies for giant sacral schwannomas.

## 5. Conclusions

The surgical management of giant sacral schwannomas remains a complex endeavor, often necessitating extensive approaches that carry the potential for significant morbidity, particularly regarding neurological function and sacropelvic stability. Critically, traditional wide resections can compromise crucial musculoskeletal attachments, leading to underappreciated long-term biomechanical deficits despite seemingly adequate neurological outcomes and pelvic ring stability.

Our novel single-posterior “Sacral Frame Technique” offers a potentially less morbid alternative by prioritizing the meticulous preservation of the lateral sacral bone margin. Leveraging the benign nature of these tumors, our technique employs an intralesional piecemeal resection facilitated by a combined trans-sacral and extrasacral approach. This strategy enables the anatomical reattachment of key pelvic stabilizers, such as the gluteus maximus muscle and the sacrotuberous and sacrospinous ligaments.

The favorable outcome observed in our presented case—characterized by complete tumor resection, early mobilization, preserved neurological function and sphincter control, and a return to pre-operative activity levels without instability at six-month follow-up—strongly suggests the potential of this bone-preserving strategy in maintaining optimal sacropelvic musculoskeletal biomechanics.

While these initial results are encouraging, further investigation through larger prospective studies with longer follow-up is warranted to definitively validate the long-term functional and biomechanical advantages of the Sacral Frame Technique compared to conventional surgical approaches. This validation will be crucial in establishing its role in optimizing patient outcomes and minimizing the often-underestimated biomechanical consequences of sacral tumor surgery.

## Figures and Tables

**Table 1:** Surgical Steps of the Sacral Frame Technique.

Perform a left gluteal arc-shaped incision with inferomedial concavity, extending towards the sacrum.
Make a midline incision of the thoracolumbar fascia.
Dissect and mobilize paravertebral muscles bilaterally.
Meticulously mobilize *en bloc* the left gluteus maximus muscle, sacrotuberous and sacrospinousligaments, coccygeus muscle.
Expose the left lateral sacral margin (from greater sciatic notch to coccyx) without anococcygealligament transection.
Identify piriformis muscle and superior gluteal artery.
Employ blunt dissection to expose the lower endopelvic/presacral tumor component.
Perform sacral laminectomy to expose the intraspinal tumor and cauda equina.
Dissect and separate the tumor from surrounding neural structures under microscopic magnification.
Identify, map (via IONM), coagulate, and section afferent nerve roots.
Meticulously dissect the lesion from the left internal iliac vein and its afferent vessels, ensuringcomplete tumor clearance.
Achieve meticulous hemostasis and place a drain.
Firmly reattach the tendinous-ligamentous complex (gluteus maximus tendon, sacrotuberous, andsacrospinous ligaments) to the preserved lateral sacral bone margin at their natural insertion site.

## Data Availability

We excluded the data availability section since our study did not report on any data present in public datasets.
